# A disease causing ATLASTIN 3 mutation affects multiple endoplasmic reticulum-related pathways

**DOI:** 10.1007/s00018-019-03010-x

**Published:** 2019-01-21

**Authors:** Laura Behrendt, Ingo Kurth, Christoph Kaether

**Affiliations:** 1 Leibniz Institut für Alternsforschung-Fritz Lipmann Institut, Beutenbergstr. 11, 07745 Jena, Germany; 2 Institute of Human Genetics, Medical Faculty, RWTH Aachen University, 52074 Aachen, Germany

**Keywords:** Atlastin, Hereditary spastic paraplegia, Endoplasmic reticulum, Secretory transport

## Abstract

Atlastins (ATLs) are membrane-bound GTPases involved in shaping of the endoplasmic reticulum (ER). Mutations in ATL1 and ATL3 cause spastic paraplegia and hereditary sensory neuropathy. We here show that the sensory neuropathy causing ATL3 Y192C mutation reduces the complexity of the tubular ER-network. ATL3 Y192C delays ER-export by reducing the number of ER exit sites, reduces autophagy, fragments the Golgi and causes malformation of the nucleus. In cultured primary neurons, ATL3 Y192C does not localize to the growing axon, resulting in axon growth deficits. Patient-derived fibroblasts possess a tubular ER with reduced complexity and have a reduced number of autophagosomes. The data suggest that the disease-causing ATL3 Y192C mutation affects multiple ER-related pathways, possibly as a consequence of the distorted ER morphology.

**Electronic supplementary material:**

The online version of this article (10.1007/s00018-019-03010-x) contains supplementary material, which is available to authorized users.

## Introduction

Length-dependent axonopathies comprise a large group of hereditary disorders affecting both afferent and efferent neurons with exceptional long axons [1, 2]. Among this large group, peripheral motor neurons are predominantly affected in Charcot–Marie–Tooth (CMT) neuropathies and distal pure hereditary motor neuropathies (HMN), whereas sensory neurons are affected in hereditary sensory and autonomic neuropathies (HSAN). Upper motor neurons, in contrast, are affected in hereditary spastic paraplegias (HSP) [3]. Common to these heterogeneous groups of neurological disorders is the length-dependent axonopathy of projecting neurons.

Causative mutations for axonopathies have been identified in genes functioning in several neuronal processes, among them intracellular transport, endosomal function and others (for review see Refs. [1, 3, 4]). Of interest are a number of axonopathy-causing genes involved in the architecture of the endoplasmic reticulum (ER). Many of them code for proteins with a reticulon-domain or reticulon-like domain, namely ARL6IP1, ATL1, ATL3, FAM134B, REEP1, RTN2 and SPAST (reviewed in Ref. [1]). The Atlastins (ATLs), gene products from *ATL1*-*3*, are large dynamin-related GTPases that are involved in ER network formation [5, 6] and maintenance [7]. ATL1 was identified as the gene product of the *ATL1/SPG3A* gene locus [8] and is mutated in app. 10% of autosomal-dominant pure/uncomplicated forms of HSP [9] and in rare cases of HSAN1 [10]. Two heterozygous mutations causing HSAN were described in ATL3, Y192C [11] and P338R [12]. Both mutations are defective in dimerization and fusion, resulting in aberrant bundling of ER tubules [13].

The role of ATLs in ER-membrane fusion is well established. ATLs can dimerize in cis and trans, and are believed to tether adjacent membranes and bring them closely together to allow fusion. ATLs can mediate fusion in vitro, but for efficient fusion additional factors were proposed [14]. Mammalian ATLs differ in tissue expression, with ATL1 being mainly expressed in the CNS [8, 15] whereas ATL2 and ATL3 are more ubiquitously expressed [16]. All ATLs localize to the ER, ATL1 throughout ER tubules, ATL2 and 3 more concentrated to three-way-junctions (3WJs) [7, 11, 17].

A role for ATLs in membrane traffic is less clear. Two publications found no role for ATL1-3 in ER–Golgi transport, assayed by knock-down, KO or overexpression of dominant negative ATLs [16, 18]. Another study concluded that ATL1 plays a role in vesicle trafficking at the ER/Golgi interface, but transport was not directly assessed [19]. Recently, it was shown that knockdown of ATL2 but less so of ATL3 in HeLa cells reduced ER–Golgi transport [20].

The pathomechanism of ATL mutations thus remains largely unclear. ATLs are directly or indirectly involved in many ER-related cellular processes like trafficking [21], ER stress [18] and lipid droplet biogenesis [22]. Many findings support the notion that mutated ATLs are defective in forming a fully functional axonal ER (reviewed in Ref. [1, 3]). Interestingly, not all HSP or HSAN disease variants of ATLs are defective in ER network formation, GTP-hydrolysis, dimer formation and fusion [23].

We here report that the ATL3 disease variant Y192C slows down ER–Golgi trafficking, induces Golgi disruption, causes ER morphology defects, reduces autophagosome formation and affects nuclear shape.

## Materials and methods

### Antibodies, plasmids and chemical compounds

See supplementary experimental procedures.

### Cloning

For construction of untagged human ATL3, ATL3 was amplified from hATL3-myc pCI neo [11] using forward (fwd) primer 5′-ggatccATGTTGTCCCCTCAGCGAGTGG-3′, introducing a* Bam*HI site and reverse (rev) primer 5′-gcggccgcCTATTGAGCTTTTTTATCCATGGATGGTCTTCC-3′, introducing a stop codon and a *Not*I restriction site. For construction of N-terminal myc-tagged human ATL3, ATL3 was amplified from hATL3-myc pCI neo using primers fwd 5′-ggatccATGGAACAAAAACTTATTTCTGAAGAAGATCTGTTGTCCCCTCAGCGAGTGG-3′, introducing a N-terminal myc-tag and a* Bam*HI site and rev: 5′-gcggccgcCTATTGAGCTTTTTTATCCATGGATGGTCTTCC-3′, introducing a stop codon and a *Not*I restriction site. In both cases, the restriction sites were used for cloning the respective fragment into pcDNA3.1 (+) Hygro. N-terminally GFP-tagged human ATL3 was constructed by amplifying ATL3 from hATL3-myc pCI neo using fwd primer 5′-CTCGAGCTATGTTGTCCCCTCAGCGAGTGG-3′, introducing a XhoI site, and rev primer 5′-ggatccCTATTGAGCTTTTTTATCCATGGATGGTCTTCC-3′, introducing a stop codon and a* Bam*HI restriction site. The restriction sites were used for cloning the fragment into pEGFP C1. The Y192C mutation was introduced by standard site-directed mutagenesis. All sequences were validated.

### Cell lines, cell culture, transfection

HeLa Kyoto (in the following called HeLa) and HuH7 cells were maintained in Dulbecco’s modified Eagle Medium + GlutaMax (Invitrogen) supplemented with 10% FBS and incubated at 37 °C, 95% relative humidity and 5% CO_2_. Cells were transfected using Lipofectamine 2000 according to manufacturer´s instruction. Primary fibroblasts from a patient with the heterozygous Y192C mutation in *ATL3* gene were derived from a 3-mm diameter punch biopsy. The procedures were approved by the local ethics committee (reference number A145/11) of the Christian-Albrechts University Medical School (Kiel, Germany). Control fibroblasts 1.1 (AG13334) and 1.2 (AG04151) were obtained from the National Institute of Ageing collection of the Coriell Cell Repository (Camden) and are from the same donor at different age. Patient fibroblasts and controls were maintained in Dulbecco’s modified Eagle Medium + GlutaMax (Invitrogen) supplemented with 20% FBS and incubated at 37 °C, 95% relative humidity and 5% CO_2_. Fibroblasts were immortalized by lentiviral infection with pCDH hTERT.

### Neuronal cell culture

Cortical neurons were isolated from murine embryonic brains (E15.5) and maintained in glia-conditioned neurobasal medium. For transfection, the calcium-phosphate method was used as described [24] with the following changes: DNA-calcium phosphate-precipitates were prepared by mixing 80 ng/µl plasmid DNA and 250 mM CaCl_2_ with equal volumes of 2 × BES buffered saline. Coverslips with attached neurons were transferred to a dish containing transfection medium (800 µM sodium pyruvate, 8 mM HEPES, 0.16% glucose, 5.5% ddH_2_O in MEM, pH 7.65). Subsequently, neurons were incubated at 37 °C for 1 h 30 min. Afterwards, neurons were incubated for 10 min in washing medium (800 µM sodium pyruvate, 8 mM HEPES, 0.16% glucose, 5.5% ddH_2_O in MEM, pH 7.35) and moved back to their original dish containing glia-conditioned medium. After 24 h, neurons were processed for immunocytochemistry.

### Immunocytochemistry and microscopy of cells

For immunofluorescence cells were grown on coverslips. After incubation, cells were fixed with 4% paraformaldehyde and processed for immunofluorescence as described [25] with antibodies as indicated. For staining of RTN4, cells were fixed with 4% paraformaldehyde containing 0.1% glutaraldehyde as described [26]. For Fig. S1a cells were fixed with 3% glyoxal at pH 5 as described [27]. For Fig. S2a cells were fixed with ice-cold MeOH for 20 min at − 20 °C. Nuclei were stained with Hoechst 33342 (Invitrogen H1399). Images were acquired on a Zeiss Axiovert200 using 20 ×, 40 × or 63 × objective and Zen2012 software. For some settings a confocal-like Apotome slider was used. Images were assembled and processed in Adobe Photoshop. Care was taken that identical settings were applied where images were to be compared. Weak signals like small vesicles or thin ER tubules were enhanced using gamma settings < 1 to match the image perception by eye. For live cell imaging, cells were plated on 3.5-cm glass bottom microwell dishes and imaged in DMEM without phenol red.

### Quantification of immunocytochemistry

The number of fluorescently stained structures within cells was quantified using ImageJ software. For this purpose, images were converted to 8-bit grayscale pictures before converting to binary pictures to distinguish objects of interest from background. Overlapping objects were separated using the ‘Watershed’ function. Finally, distinct particles were counted using the ‘Analyze particle’ function. Y192C-expressing HuH7 cells were smaller in size, therefore, α-tubulin staining and CellProfiler were used to quantify and normalize cell size. For quantification of the Golgi fragmentation index, the relative frequency distribution of the number of fragments per cell for each condition was tabulated and the index was calculated using a published formula [28].

### Cell lysis, western blotting, deglycosylation assay

Cells were lysed in STEN buffer, separated by SDS-PAGE, transferred to PVDF membranes and probed with antibodies as described [29]. Deglycosylation of VSVG-EYFP was performed basically as described [29] but using direct cell lysates instead of immunoprecipitates.

### Autophagy assay

Cells seeded on cover slips were transfected with respective plasmids for 6 h followed by medium change. After 24 h, cells were treated with control medium or pretreated with medium containing 50 μM chloroquine (CQ) for 1 h followed by starvation medium [30] with 50 μM CQ for 3 h. Finally, cells were fixed and processed for immunocytochemistry.

### ER stress assay

HeLa cells were seeded in 6 well plates and transfected the next day with respective plasmids for 6 h, followed by medium change. 24 h after transfection, cells were treated with 2.5 µg/ml tunicamycin or DMSO for 24 h, lysed and processed for western blotting.

### SEAP assay

For measuring secretion activity HeLa cells stably expressing SEAP were used as described [31].

### Stain-free gel imaging and protein quantification

Stain-free gel imaging was used to assess total protein levels (loading control). After separation, gels were soaked in 10% TCA solution for 5 min followed by three washing steps with dH_2_O. Subsequently, gels were imaged using the Gel Doc System (Bio-Rad). The intensity of whole lanes was quantified after background subtraction using the Rolling Ball algorithm in ImageJ. The obtained intensity values were used to normalize the western blot signals of proteins of interest. As described by others [32, 33] we found this method superior to normalization to actin or tubulin or other house-keeping genes.

### Statistics

Reported values represent mean ± SEM, unless stated otherwise. Statistical differences between the means of two groups were determined using Welch’s *t* test, unless stated otherwise. Numbers of independent replicates and *p* values are indicated in figures or figure legends, a *p* value < 0.05 was considered significant.

## Results

### ATL3 Y192C disrupts the ER network and deforms the nucleus

**Fig. 1:**
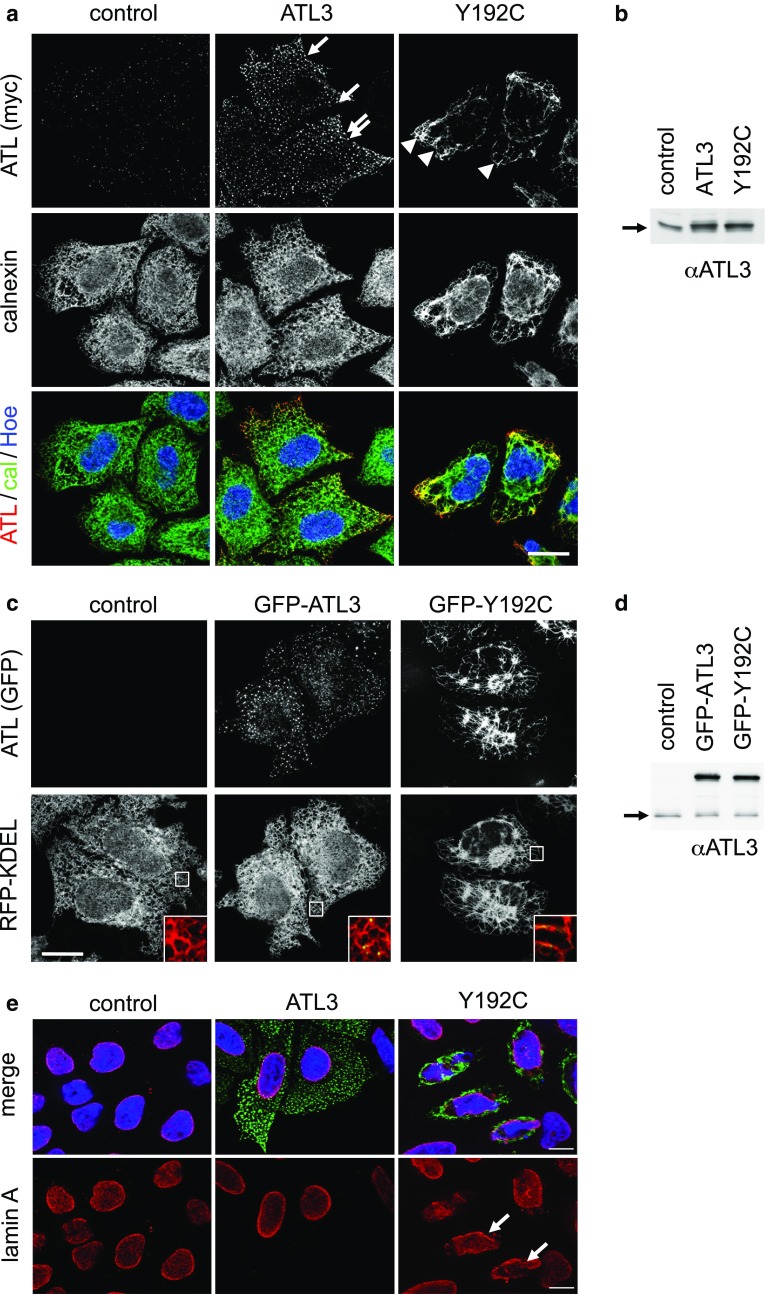
ATL3 Y192C induced ER-morphology changes and malformation of the nucleus. **a** HeLa cells were transfected with a control vector, ATL3-myc or ATL3 Y192C-myc, fixed and stained with antibodies against calnexin and myc. Arrows indicate punctate ATL3 localization, arrowheads ATL3 Y192C mislocalization at distorted ER. **b**, **d** HeLa cells transfected as indicated were lysed and processed for immunoblotting with anti-ATL3 antibodies. The arrows indicate endogenous ATL3. Equal loading was validated by stain-free gel-imaging (see Fig. S6, also for full-size blots). **c** HeLa cells were transfected with pplss-KDEL-mRFP (RFP-KDEL) as ER marker and GFP-ATL3 or GFP-ATL3 Y192C and analyzed by live cell imaging. Lower panel, magnifications of the boxed areas. **e** HeLa cells were transfected with a control vector, ATL3-myc or ATL3 Y192C-myc, fixed and stained with antibodies against myc and lamin A. Arrows point to distorted nuclei. **a**, **c**, **e** Single Apotome sections of representative images from *n* = 3 independent experiments. Nuclei in **a**, **c** were stained with Hoechst 33342, scale bar 10 μm

ATL3 is expressed ubiquitously [16]. We, therefore, argued that general cellular functions of ATL3 can be analyzed in cell lines. To this end, we transfected myc-tagged ATL3 or the HSAN-causing mutation ATL3 Y192C in HeLa cells and analyzed their subcellular localization (Fig. 1a). As published before [11], ATL3 localizes predominantly to ER 3WJs, whereas the Y192C variant is distributed along a misshaped ER with much less 3WJs. Untagged as well as N- and C-terminally myc-tagged ATL3 localize to 3WJs (Figure S1a), confirming previous reports [7, 11, 17] and demonstrating that the tag does not interfere with localization. Expression levels of ATL3 and ATL3 Y192C are comparable, suggesting that no differences in expression levels cause the different phenotypes (Fig. 1b). We next analyzed the ER morphology by live-cell microscopy using GFP-ATL3 variants (Fig. 1c). While ATL3 overexpression does not change the extensive tubular ER-network, the Y192C mutation had a strong impact on the ER. ER network complexity was strongly reduced with elongated tubules and fewer 3WJs. The GFP-tag is tolerated only at the N-terminus, not the C-terminus of ATL3. ATL3-GFP does not localize to 3WJs, but is more broadly distributed in the ER, suggesting that only small tags at the C-terminus are tolerated (Fig. S1c). A similar ER morphology change is induced by overexpression of the corresponding mutation in ATL1, Y196C (Fig. S2a). Overexpression of ATL3 Y192C did not affect the microtubule network (Fig. S2b). It did also not cause up-regulation of BIP, suggesting the overexpression of the mutant ATL3 does not cause ER stress (Fig. S2c, d). Interestingly, we noted a deformation of the nucleus in most ATL3 Y192C-transfected cells (83% deformed nuclei ± 0.04 vs. 8% ± 0.03 in ATL3-transfected cells; SEM, *n* = 3; 224 and 369 cells, respectively; Fig. 1e).

### ATL3 Y192C delays ER export by reducing the number of ERES

**Fig. 2:**
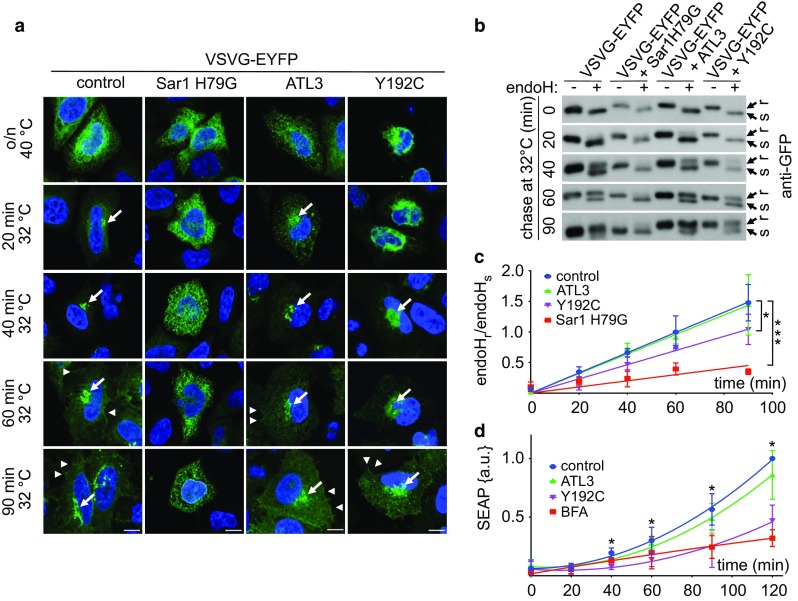
ATL3 Y192C delayed ER to Golgi transport. HeLa cells were transfected with VSVG-EYFP and a control vector, Sar1 H79G, ATL3-myc or ATL3 Y192C-myc, incubated overnight at 40 °C and chased at 32 °C for indicated times, followed by fixation (**a**) or cell lysis (**b**). **a** Fixed cells were stained with an antibody against myc to identify transfected cells. Nuclei were stained with Hoechst 33342. Images represent single Apotome sections. Arrows indicate Golgi localization of VSVG-EYFP, arrowheads VSVG-EYFP localization at the plasma membrane. For clarity only EYFP staining is shown. Scale bar, 10 μm, *n* = 3 independent experiments. **b** Lysates were digested with or without endoH, separated using SDS-PAGE, blotted and probed with an antibody against GFP. Stain-free gel imaging was used as loading control (Fig. S6, see also for full-size blots). **c** Quantification of the ratio of the upper, endoH resistant and the lower, endoH sensitive band. Signals were quantified using ImageJ. Data points were fitted using GraphPad Prism’s linear regression function. Data are displayed as mean ± SD, *p < 0.05 (one-way ANOVA followed by Tukey’s multiple comparisons test, slopes of each linear regression function were compared). *n* = 3 independent experiments. **d** HeLa cells stably expressing SEAP were transfected with a control vector or ATL3-myc or ATL3 Y192C-myc or treated with BFA (0.1 μg/ml). After the indicated times, SEAP activity was determined, data normalized against the obtained maximum and minimum value and fitted using GraphPad Prism’s polynomial second order function. Data are displayed as mean ± SD, *p < 0.05, ***p < 0.001, Welch’s *t* test, *n* = 4 independent experiments

To test whether ATL3 Y192C affects export from the ER we made use of the temperature-sensitive tsO45 mutant VSVG [34] tagged with EYFP (VSVG-EYFP) [35]. This trafficking reporter is misfolded at 40 °C and consequently accumulates in the ER. Upon shifting to the permissive temperature (32 °C), VSVG-EYFP folds properly, exits the ER and travels to the plasma membrane [36, 37]. Using its N-glycosylation sites the localization can be assessed with the help of endoglycosidaseH (endoH), because ER-localized VSVG-EYFP is endoH-sensitive whereas Golgi and beyond-localized VSVG-EYFP is endoH resistant [31]. In HeLa cells transfected with ATL3 or ATL3 Y192C together with VSVG-EYFP only the Y192C mutation, but not the wild type, slowed down ER–Golgi transport (Fig. 2a–c). Overexpression of the dominant-negative Sar1 H79G mutant [38] served as positive control for transport inhibition. Linear regression analysis and slope determination indicated that ATL3 Y192C expression compared to ATL3 slowed down the acquirement of endoH resistance by app. 25% (slope: control 0.017 ± 0.0003; Sar1 H79G 0.005 ± 0.0008; ATL3 0.016 ± 0.0005; ATL3 Y192C 0.012 ± 0.0006). The findings were corroborated by measuring the secretion rate of a secreted alkaline phosphatase (SEAP) [39], which was also reduced in ATL3 Y192C expressing cells compared to ATL3 (Fig. 2d). BFA [40] served as positive control. The data suggest that ATLs are directly or indirectly involved in ER export, possibly via their influence on ER morphology.

**Fig. 3:**
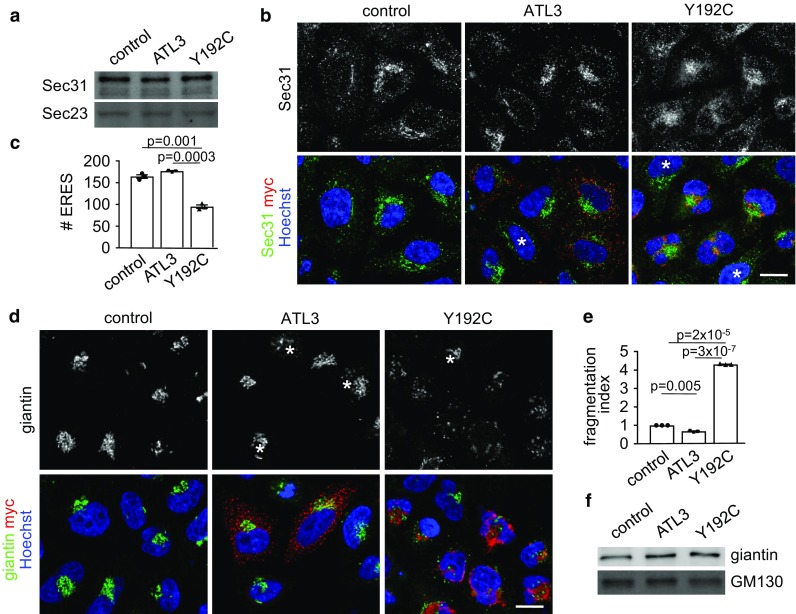
ATL3 Y192C reduced the number of ERES and fragmented the Golgi. HeLa cells were transfected with a control vector or ATL3-myc or ATL3 Y192C-myc, incubated for 24 h, lysed (**a**, **f**) or fixed and processed for immunofluorescence with indicated antibodies and Hoechst 33342 to label nuclei (**b**, **d**). **a** Lysates were blotted and probed with antibodies against Sec31 and Sec23. *n* = 3 independent experiments. **b** Single Apotome sections of transfected HeLa cells as indicated. Representative images of 3 independent experiments are shown, scale bar, 10 μm. **c** Quantification of **b**. Sec31 labeled ERES were counted using ImageJ, for example images see Fig. S4. Analyzed were 66–120 cells per condition from *n* = 3 independent experiments. **d** Transfected cells were stained with antibodies against giantin and myc. **b**, **d** Asterisks indicate untransfected cells, scale bar 10 μm. **e** Quantification of **d**. Golgi fragments were counted using ImageJ and the fragmentation index calculated. Analyzed were 115 cells per condition from three independent experiments. **f** Lysates were blotted and probed with antibodies against giantin and GM130. *n* = 3 independent experiments. **a**, **f** For full size blots and stain-free gel imaging as loading control see Fig. S6. **c**, **e** Values represent mean ± SEM, *p* values are indicated (Welch’s *t* test)

To analyze the underlying cause for this reduced export capacities we probed cell lysates for the presence of Sec23 and Sec31, two COPII components of the molecular machinery involved in ER export [41]. No difference in expression levels of both was observed in ATL3 or ATL3 Y192C expressing cells (Fig. 3a). However, in ATL3 Y192C expressing cells the number of ER exit sites (ERES) as identified with two different ERES-components, Sec31 (Fig. 3b, c; Fig. S4) and Sec16 (Fig. S3b, c, Fig. S4) was reduced by around 45%. The reduced ER export rate could affect the ER–Golgi intermediate compartment (ERGIC), a very dynamic organelle between the ER and the Golgi (reviewed in Refs. [42, 43]). Indeed, ATL3 Y192C but not ATL3 transfection in Hela cells resulted in reduced ERGIC53 staining (Fig. S3a). ERGIC53 is a marker of the ERGIC [44]. In addition, the typical ERGIC distribution in a Golgi-like, juxtanuclear accumulation and numerous peripheral vesicles was transformed to a broader distribution partially colocalizing with the distorted ER and loss of the peripheral vesicles (Fig. S3a). This suggested a disturbed recycling of ERGIC components and partial retention in the ER due to the reduced ER-export. We next analyzed the Golgi complex, because reduced transport to the Golgi may result in morphological changes like fragmentation, often observed in neurodegenerative diseases (reviewed in Refs. [45, 46]). Indeed, expression of ATL3 Y192C, but not ATL3 resulted in a fragmentation of the Golgi, as indicated by staining the Golgi with an antibody against giantin (Fig. 3d) and by determining a Golgi fragmentation index (Fig. 3e). The Golgi fragmentation is not caused by a disturbed microtubule network (Fig. S2b). Expression levels of two Golgi proteins, GM130 and giantin, remained unaffected (Fig. 3f).

Taken together, the data suggest that by reducing the number of ERES, ATL3 Y192C causes a reduced transport of cargo from the ER, resulting in disruption of the ERGIC and fragmentation of the Golgi.

### ATL3 Y192C reduces the formation of autophagosomes

**Fig. 4:**
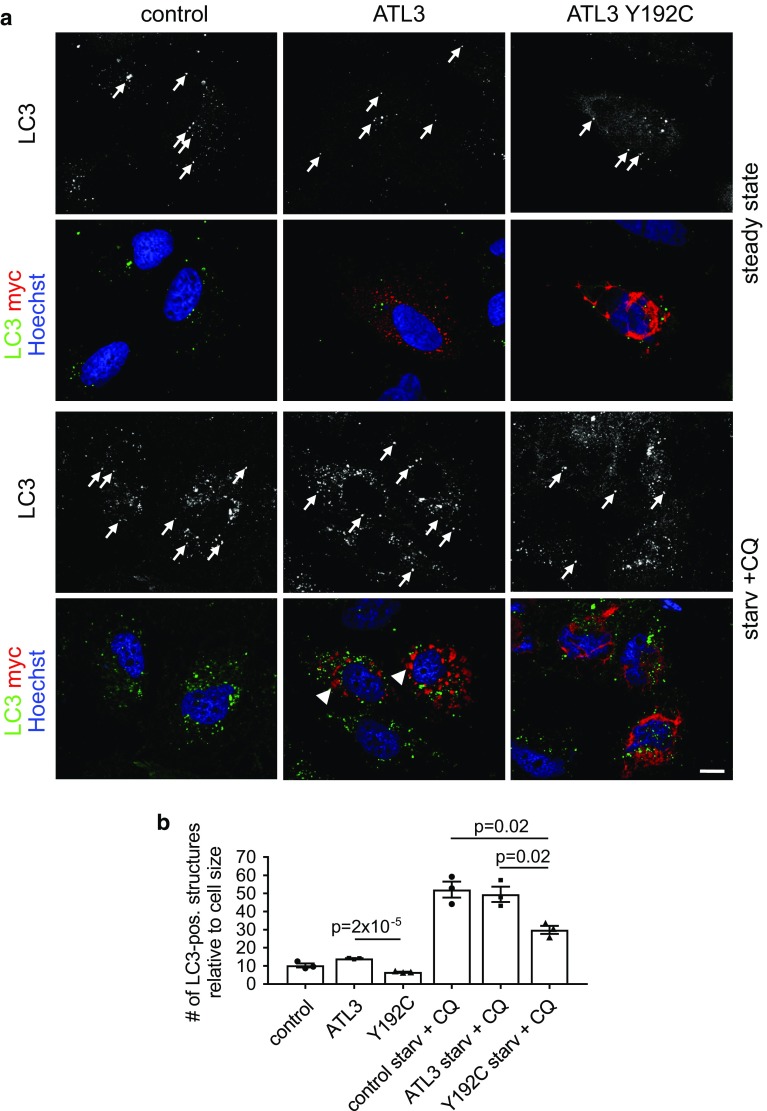
ATL3 Y192C affects autophagosome formation. HuH7 cells transfected with empty vector (control), ATL3-myc or ATL3 Y192C-myc were incubated for 24 h, treated without (steady state) or with starvation medium plus 50 μM chloroquine (starv. + CQ) for 3 h, fixed and processed for immunofluorescence staining with antibodies against LC3 and myc and with Hoechst 33342 to label nuclei. **a** Single Apotome sections of representative cells from *n* = 3 independent experiments. Arrows, LC3-positive vesicles. Scale bar 10 μm. Note that the starvation medium causes ER morphology changes, indicated by the relocalization of ATL3 to larger aggregates (arrowheads). **b** Quantification of cells from **a**. Displayed is the average number of LC3-positive vesicles per cell normalized to the cell size. Error bars depict SEM. Analyzed were 26–30 cells for each condition from three independent experiments, *p* values are indicated (Welch’s *t* test)

The ER plays an important role in autophagy, and ERES are key players in autophagosome formation (reviewed in Ref. [47]). We, therefore, studied whether ATL3 Y192C may affect autophagy by quantifying the number of LC3-positive structures. LC3 in its lipidated form is a marker for autophagosomes [48]. Expression of ATL3 Y192C but not ATL3 in HuH7 cells significantly reduced the number of LC3-positive structures in untreated as well as in starved cells treated with chloroquine (Fig. 4a, b). This suggests that the ER morphology changes and/or the reduced ERES number induced by ATL3 Y192C severely affects autophagosome formation or processing.

### ATL3 Y192C is excluded from axonal ER

**Fig. 5:**
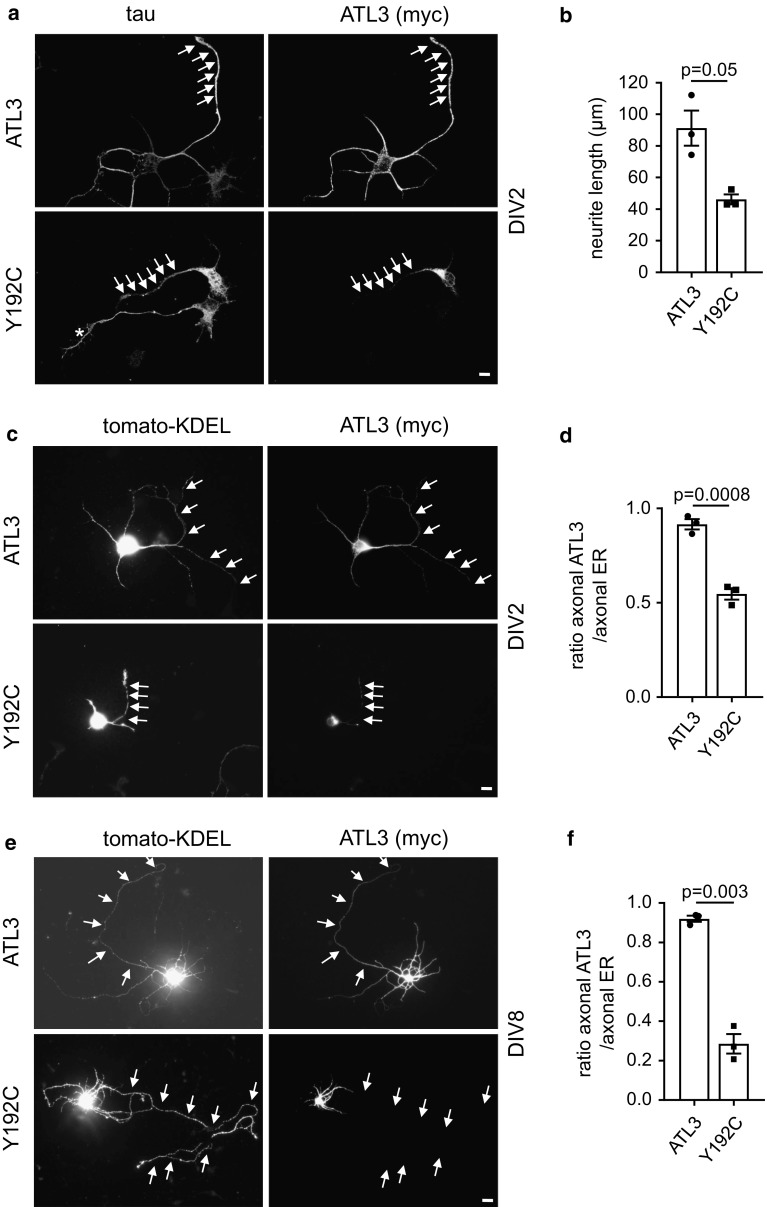
Axonal outgrowth is inhibited and ATL3 Y192C is mislocalized in cultured neurons. Cortical murine primary neurons were transfected with myc-tagged ATL3 variants (**a**, **b**) or additionally co-transfected with tomato-KDEL as ER-marker (**c**–**f**), fixed 24 h later and stained with antibodies as indicated. The DIV at day of fixation is indicated. Arrows indicate axons. **a** Note the untransfected neuron just below the transfected one with a normally grown axon (asterisk). **b** Quantification of **a**. Displayed is the mean length of the longest neurite (the future axon). Error bars depict SEM. **d**, **f** Quantification from **c**, **e**. The ratio depicts the mean length of ATL3 staining divided by the mean length of axonal ER staining staining extending into the axon. **a**–**f** Analyzed were 32–51 neurons for each condition from three independent experiments. Error bars depict SEM, *p* values are indicated (Welch’s *t* test). Scale bar 10 μm

We next wanted to analyze the ATL3 Y192C mutation in neurons. To this end, we transfected ATL3 or ATL3 Y192C in DIV1 (1 day in vitro) murine cultured cortical neurons and analyzed them 24 h later (DIV2). In DIV2 neurons ATL3 is distributed throughout the longest neurite (the future axon) and the shorter neurites, whereas ATL3 Y192C is mostly restricted to the cell body and not extending into neurites (Fig. 5a). The overexpression of ATL3 Y192C strongly impairs the outgrowth of the future axon, reducing the mean length of the longest neurite by 50% (Fig. 5b). Interestingly, in ATL3 Y192C expressing cells the axon still contains ER as indicated by a fluorescent ER marker, tomato-KDEL (Fig. 5c, d) or endogenous RTN4 (not shown), suggesting a localization/transport defect of mutant ATL3 but not a collapse of the axonal ER. To test if this effect would be also observed in older, more mature neurons with already established long axons, we transfected DIV7 cultured neurons with ATL3 wt and Y192C mutant and tomato-KDEL (Fig. 5e, f). In ATL3 expressing neurons the ATL3 staining extends into the axon almost as far as the ER marker. In contrast, in ATL3 Y192C expressing neurons the mutant ATL3 only extends into proximal axon segments, whereas tomato-KDEL also is present in distal axons (Fig. 5f). This suggests that mutant ATL3 Y192C cannot properly localize to the axonal ER and that this lack of axonal ATL3 contributes to disease pathology.

### ATL3 Y192C patient cells display a reduced ER network complexity and are compromised in autophagy

**Fig. 6:**
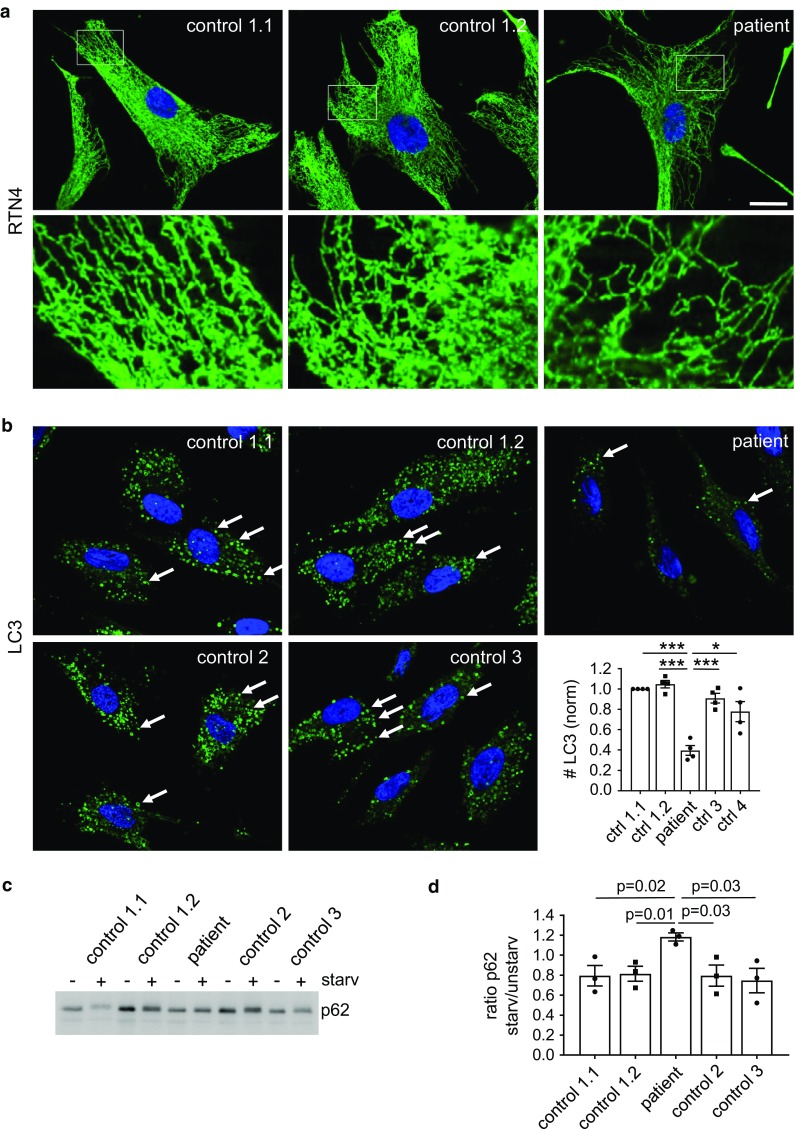
Reduced ER complexity and deficits in autophagy in patient cells. Fibroblasts derived from healthy donors (control) and a HSAN1 patient with ATL3 Y192C mutation (patient) were incubated for 24 h and **a** fixed and processed for immunofluorescence staining with antibodies against RTN4 or **b** treated with starvation medium plus 50 μM chloroquine (starv. + CQ) for 3 h. Thereafter cells were fixed and processed for immunofluorescence staining with LC3 antibodies and with Hoechst 33342 to label nuclei. Arrows, LC3-positive vesicles. Graph: displayed is the ratio of LC3-positive vesicles per cell normalized to control 1.1. Error bars depict SEM, *p = 0.001; ***p = 2 × 10^−5^ to 2 × 10^−4^ (Student’s *t* test). At least 177–239 cells were analyzed for each condition from *n* = 4 independent experiments. **c** Control and patient fibroblasts were treated with or without starvation medium for 3 h, lysed, blotted and probed for p62. For full size blots and stain-free gel imaging as loading control see Fig. S6. **d** Quantification of *n* = 3 independent experiments from **c**. Displayed is the mean ratio of p62 intensity in starved/unstarved conditions. Error bars depict SEM. *p* values are indicated (Student’s *t* test). Single Apotome sections, scale bar 10 μm

To analyze the effect of ATL3 mutations under pathophysiological conditions, we studied immortalized fibroblasts from an individual harboring the Y192C mutation and healthy controls (Fig. 6). Compared to two fibroblast cell lines derived at different age from a healthy donor, the patient fibroblasts have a less complex tubular ER network (Fig. 6a). For investigation of autophagic vesicles two additional fibroblast lines from healthy donors were compared to the patient cell line. Corroborating the results in transfected HeLa cells, the number of steady-state as well as starvation-induced LC3-positive vesicles was significantly reduced in patient cells compared to all four controls (Fig. 6b). p62/sequestosome1 is an autophagy receptor that is degraded in autophagic conditions and increased upon inhibition of autophagy [49]. Consequently, upon autophagy stimulation p62 levels were reduced in the four control fibroblast lines, but increased in ATL3 Y192C fibroblasts, indicating a compromised autophagy (Fig. 6c, d). All fibroblast lines expressed similar levels of ATL1-3 (Fig. S5). Taken together, also patient cells show a compromised ER and defects in autophagy, confirming that the results obtained by overexpression mimic, albeit exaggerate, the patient situation.

In sum, the data suggest that ATL3 Y192C causes numerous defects in ER and Golgi morphology, ER-exit, autophagy and neurite outgrowth.

## Discussion

Causative heterozygous mutations for HSP (SPG3A) and HSAN were identified in ATL1 and ATL3, respectively [8, 11, 12]. Both ATL1 and ATL3 are ER-shaping membrane proteins, but the precise pathomechanism has not been elucidated. We here show that the HSAN-causing ATL3 mutation Y192C but not ATL3 affects multiple ER-related pathways when overexpressed in cell lines or cultured neurons. The ATL3 Y192C is a dominant negative mutation [11], justifying the use of an overexpression paradigm to study the role of the mutation, although this does not fully match the patient situation. However, since in all our assays only the overexpression of ATL3 Y192C but not ATL3 caused a phenotype we are confident that the observed effects are indeed due to the mutation and not the overexpression per se. In addition, using fibroblasts from a HSAN patient carrying the Y192C mutation heterozygously, we could confirm that features we detected by transfection (ER morphology, autophagy defects) are similarly observed in the heterozygous patient cells. The overexpression approach most likely allows detection of cellular dysfunctions that may go unnoticed in patient fibroblasts in vitro. Subtle changes in, for example, ER–Golgi transport might be difficult to detect, but still be relevant for disease progression. The age of onset of HSAN1 caused by ATL3 Y192C is 14–30 years and only sensory neurons are affected [11], despite the wide-spread expression of ATL3. This indicates that cellular dysfunctions are subtle in patients and may need to be exaggerated in in vitro studies to detect them, but it should be kept in mind that the overexpression does not exactly mimic the patient situation.

Why are neurons with their extremely long axons preferentially affected? We speculate that their proteostasis systems might be simply already at their limits without further reserves, just coping with the load at young age. Any further burden imposed by aging of the neuron in conjunction with the mutation then may lead to neurodegeneration. Less polarized cells may have more reserves (and in fact in most cases a much shorter lifespan) and therefore, remain asymptomatic.

Using sensitive assays for ER–Golgi transport (with VSVG-EYPF) and secretion (with SEAP) we detected effects of the disease-causing ATL3 Y192C variant on ER export. The reduced secretion is caused by a reduction in the number of ERES, and this also affects the structure of the ERGIC and the Golgi, probably as a consequence of the reduced ER-export. Indeed, ERGIC53, a marker for the ERGIC, seems to accumulate in the ER due to its reduced export. In contrast, using overexpression of dominant-negative ATL1-3, Rismanchi et al. found no effect on transport of VSVG-GFP to the plasma membrane [16]. However, the transport was not quantified and small changes might have gone unnoticed. Namekawa et al. indirectly concluded from changes in ER/Golgi morphology that ATL1 HSP-mutations affected trafficking, but no transport was analyzed [19]. In addition to the transport defects we observed a strongly compromised ER-network, reduced number of autophagosomes and increased p62-levels, defects in nuclear shape and neurite outgrowth deficits in primary neurons.

Are all the observed cellular changes directly caused by ATL3 Y192C, or are they indirectly the consequence of a single primary deficit? This is difficult to answer at this point. ATLs have mainly been associated with ER-network formation [5, 6] and a convincing model how ATLs mediates fusion was published [50]. The ATL3 Y192C mutation also causes severe deficits in establishing an ER network, up to total loss of tubules in highly overexpressing cells (this work, [11]). While this manuscript was in preparation, it was reported that the ATL3 Y192C mutation is defective in ER fusion and results in aberrant tethering of ER tubules [13]. It is conceivable that for proper assembly and function ERES need an exactly defined ER structure with the right degree of curvature and sheet/tubule ratio [51]. A condensed, non-branched ER induced by ATL3 Y192C would simply not provide enough suitable assembly sites for ERES components, resulting in reduced ER-export. The reduced numbers of ERES could also explain the deficits in autophagy, since ERES are tightly linked to the formation of autophagosomes [52, 53], and the axonal outgrowth deficits. Our findings on autophagy are in contrast to a recent report in which increased autophagy flux was observed in HeLa cells expressing ATL3 Y192C, while patient cells were not analyzed [54]. This discrepancy remains unclear and may be attributed to different cellular systems or levels of overexpression. Finally, the nuclear shape malformations we detected might be indirectly induced by the condensed juxtanuclear ER. Interestingly, ATLs via their function in maintaining a proper ER topology, sustain the efficient targeting of proteins to the inner nuclear membrane [20]. Maybe the nuclear malformations are a consequence of compromised transport to the inner nuclear membrane.

On the other hand, it is conceivable that ATLs mediate not only the fusion of ER tubules but have additional direct functions in the pathways affected here. This is supported by the study of Ulengin et al. who analyzed many ATL1 HSP-mutations and categorized them in two classes: one class comprised mutations with a reduced GTPase activity that had deficits in dimerization and membrane fusion, the other one mutations that had no obvious effect in the respective assays [23]. Additional functions of ATLs could encompass a direct role in ERES formation and/or a direct role in autophagosome formation.

ATL3 Y192C caused neurite growth defects in young neurons and interestingly was not present in distal axons in DIV2 and DIV8 cultured neurons. This cannot be explained by non-establishment or retraction of distal axonal ER or a non-continuous ER. Soluble KDEL-tagged ER-marker is distributed throughout the axon in ATL3 Y192C expressing neurons, indicating a continuous ER ranging into distal axons. It remains to be shown to what extend the ultrastructure of the ATL3-free axonal ER in ATL3 Y192C expressing neurons is changed and to what extend this contributes to pathology. How the mutant ATL3 is prevented from localizing to distal axons remains an open question, maybe involving unknown immobilization/segregation mechanisms.

Why does a mutation in a homologous position (Y196C in ATL1, Y192C in ATL3) of two very conserved proteins cause different diseases? One explanation could be different expression patterns, with ATL1 being more expressed in motor neurons and ATL3 in sensory neurons. Another explanation could be that ATLs have overlapping, but not identical functions. ATL3 is more concentrated in 3WJs, whereas ATL1 is more evenly distributed along the ER [7, 11, 17]. This was suggested to be the consequence of a slower GTPase activity of ATL3 [17], pointing to differences between the molecules. Endogenous ATLs do not seem to form heterodimers [16], but if this is true for disease-causing mutated proteins in patient cells has to be investigated. Single or double knock-down/knock-out of one or two of ATL1-3 has little effect on ER morphology [18, 20]. Does this suggest that ATL1 Y196C and ATL3 Y192C are forming heterodimers with other ATL family members, explaining why they act dominant-negatively? During revision of this manuscript Liang et al. demonstrated that exogenously expressed ATL2 interacted with endogenous ATL3 [55], but further work is needed to fully address this point.

Future studies will also tell if one or more of the observed cellular deficits are causative for the axonal degeneration. A careful assessment and comparison of mutations in ATL1 and 3 but also of mutations in other ER-shaping HSP- or HSAN-genes with as many assays as possible might reveal commonalities or differences that point to the cellular pathway(s) whose malfunctions are actually responsible for the disease.

## Electronic supplementary material

Below is the link to the electronic supplementary material.

Fig. S1: The position of the myc-tag does not change localization of ATL3. a) C- or N-terminally myc-tagged or untagged ATL3 were transfected in Hela cells, glyoxal-fixed for optimal ER morphology preservation, immunostained with myc- or ATL3-antibodies as indicated and imaged with Apotome microscopy, displayed are single sections. White boxes indicate position of enlarged images. Arrows indicate three-way junctions. Note that the ATL3 antibody recognizes only transfected ATL3, not endogenous. b) Lysates of cells transfected as in a) were processed for Western Blotting with anti-ATL3 antibodies. For full size blots and stain-free gel imaging as loading control see Fig. S7. c) A C-terminal EGFP-tag interferes with ATL3 localization. Hela cells transfected with ATL3-myc or ATL3-EGFP were fixed and processed for immunostaining with anti-myc antibody (right) or directly mounted (left) and imaged by fluorescence microscopy. Shown are representative cells with moderate expression levels of exogenous ATLs. Localization pattern is identical in cells with barely detectable expression. Scalebar in (a,c) 10 μm, nuclei stained with Hoechst 33342.

Fig. S2: ATL1 Y196C causes ER network disruption and ATL3 Y192C does not influence the microtubule network and does not cause ER stress. a) HeLa cells transfected with C-terminally myc-tagged ATL1 variants as indicated were fixed after 24h and stained for calnexin and myc and DNA stained by Hoechst. Apotome-sections, scale bar 10 µm. b) HeLa cells transfected with empty vector (control) or C-terminally myc-tagged ATL3 variants as indicated were fixed 24h later in MeOH and stained for tubulin and myc. Nuclei are stained by Hoechst 33342. Deconvoluted Apotome-sections, scale bar 10 µm. No change in the typical, radial microtubule network is visible, independent of ATL3 or ATL3 Y192C expression. c) HeLa cells transfected with empty vector (control) or C-terminally myc-tagged ATL3 variants as indicated were lysed and probed for BIP. Cells were treated with 2,5 µg/ml tunicamycin (tun) for 24h as positive control. Only the tunicamycin-treated cells have up-regulated BIP levels. For full size blots and stain-free gel imaging as loading control see Fig. S7. d) BIP levels from n=3 independent experiments like in c) were quantified, the mean expression level of control-transfected cells set to 1 and the other values related to that. Error bars represents SEM, p-value is indicated (Welch’s t-test).

Fig. S3: a) ATL3 Y192C expression changes the distribution of the ERGIC. HeLa cells transfected with ATL3-myc or ATL3 Y192C-myc were fixed after 24 h and stained with anti-ERGIC53 and anti-myc antibodies. Asterisks depict untransfected cells, arrows point to spread, abnormal ERGIC53 distribution. b) ATL3 Y192C reduces the number of Sec16-positive ERES. HeLa cells were transfected with ATL3-myc or ATL3 Y192C-myc, incubated for 24h, fixed and processed for immunofluorescence with indicated antibodies. The asterisk depicts an untransfected cell. a,b) Single Apotome sections, nuclei stained with Hoechst 33342, scalebar 10 μm. c) Quantification of cells from (b) using ImageJ, see Fig. S4. Analyzed were 90-95 cells for each condition from 3 independent experiments each. Depicted is the mean number of ERES, error bars depict SEM, p-values are indicated (Welch’s t-test).

Fig. S4: Examples for ERES quantification. HeLa cells transfected with ATL variants as indicated were fixed and processed for Sec31 and Sec16 immunofluorescence, respectively. Shown are the images from Fig. 3 and S3 before (top rows in a,b) and after binarization and counting of ERES using ImageJ (bottom rows in a,b). The indicated numbers refer to the number of ERES in the displayed cells.

Fig. S5: a) Human fibroblasts express similar levels of ATL1-3. Fibroblasts were lysed and probed for indicated antibodies. b) Specificity of ATL2 antibody as indicated by Western Blot of human fibroblast lysates transfected with control or ATL2-specific siRNA. Asterisks in (a,b) indicate unspecific bands. For full size blots and stain-free gel imaging as loading control see Fig. S7.

Fig. S6: Full-size blots and stain-free gel imaging of immunoblots from Figs 1-6. Note that for Sec23/31 and giantin immunoblotting the same membrane or gel, respectively, was used.

Fig. S7: Full-size blots and stain-free gel imaging of immunoblots from Figs S1,2,5.

Supplementary material 1 (PDF 3880 kb)

Supplementary material 2 (DOCX 19 kb)
