# Supplementary figures and images for: A disease causing ATLASTIN 3 mutation affects multiple endoplasmic reticulum-related pathways
Source: Cell Mol Life Sci. 2019 Jan 21;76(7):1433–45. doi: 10.1007/s00018-019-03010-x (PMC6420906; doi:10.1007/s00018-019-03010-x)

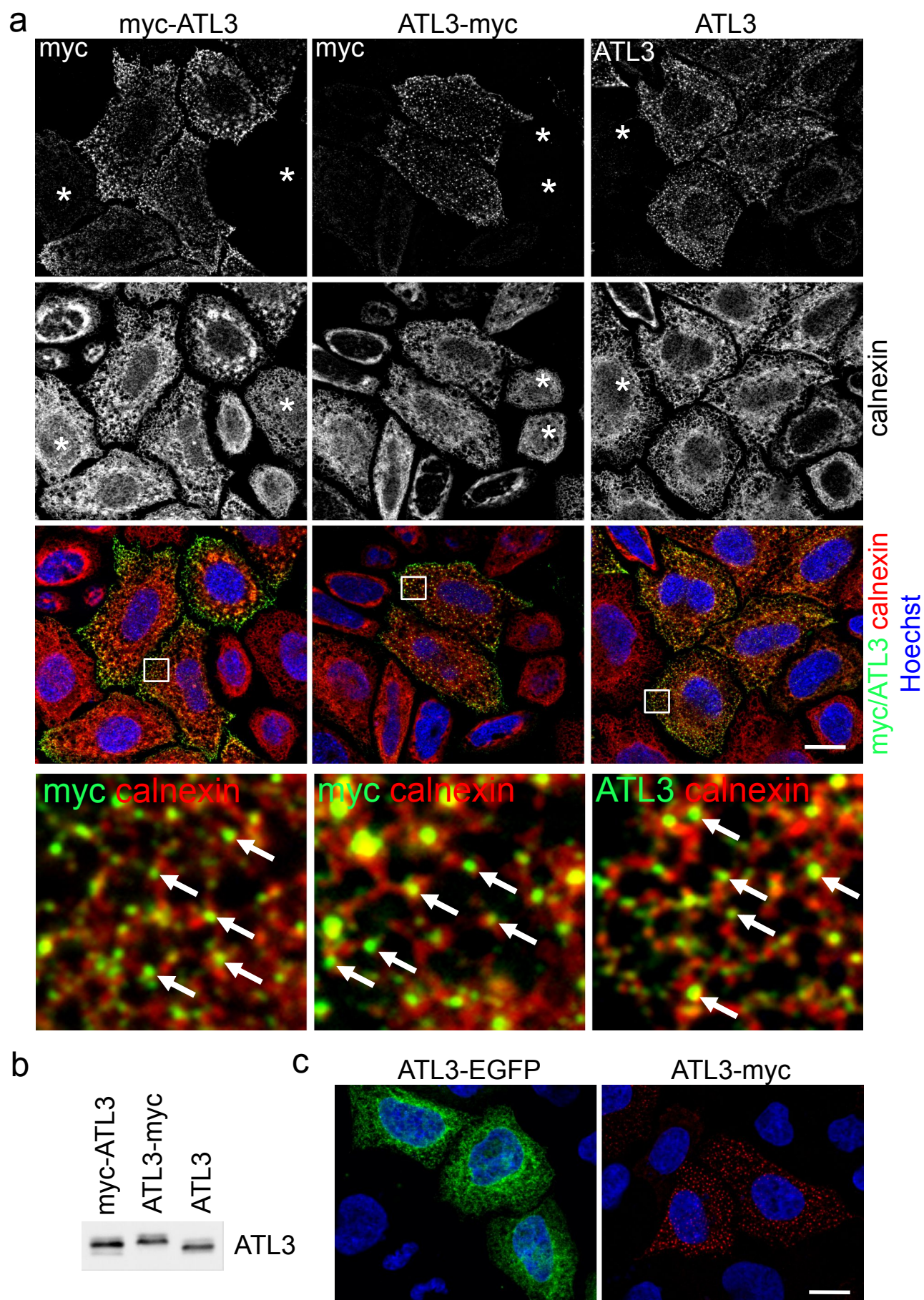

Fig. S1

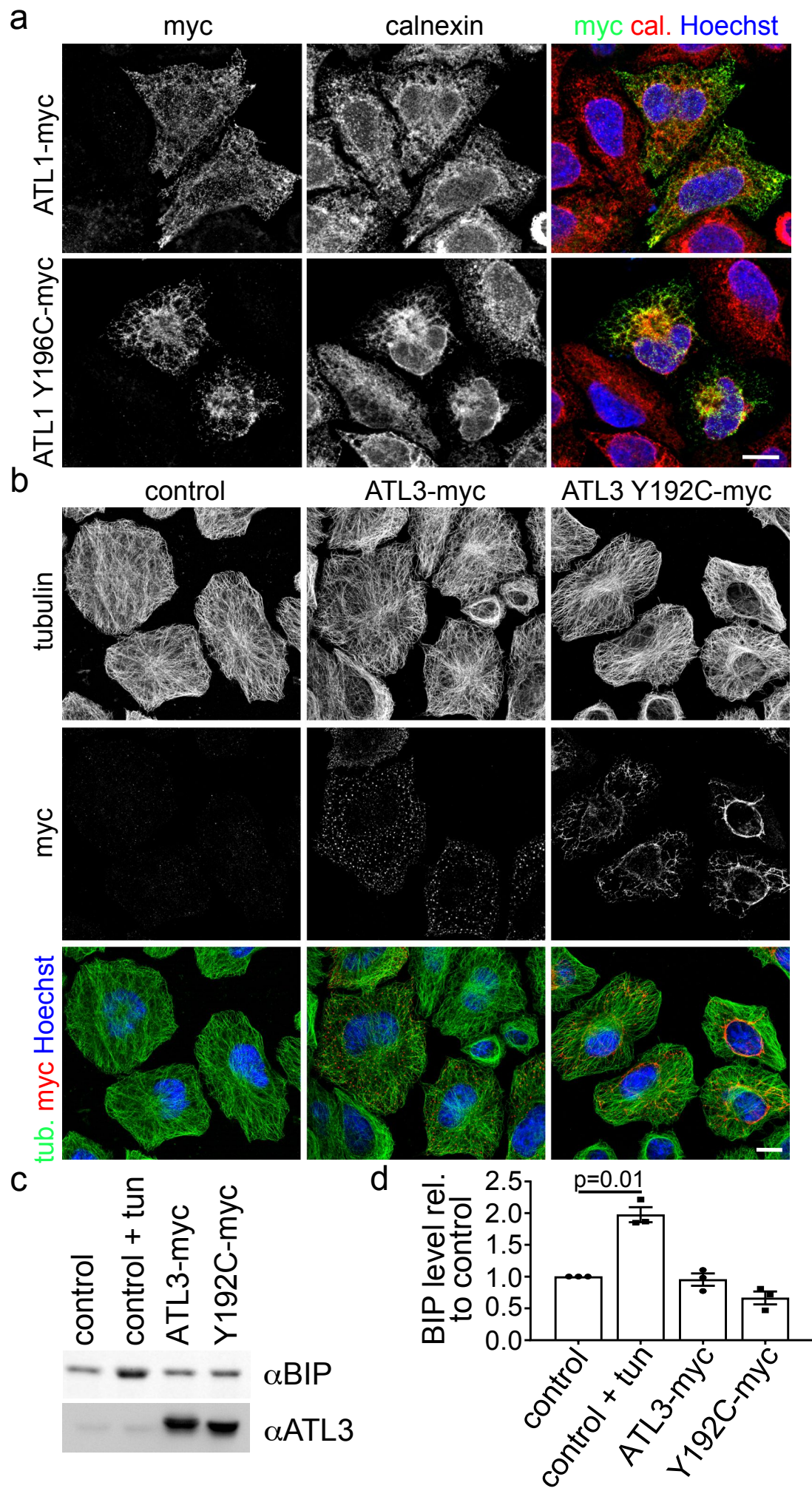

Fig. S2

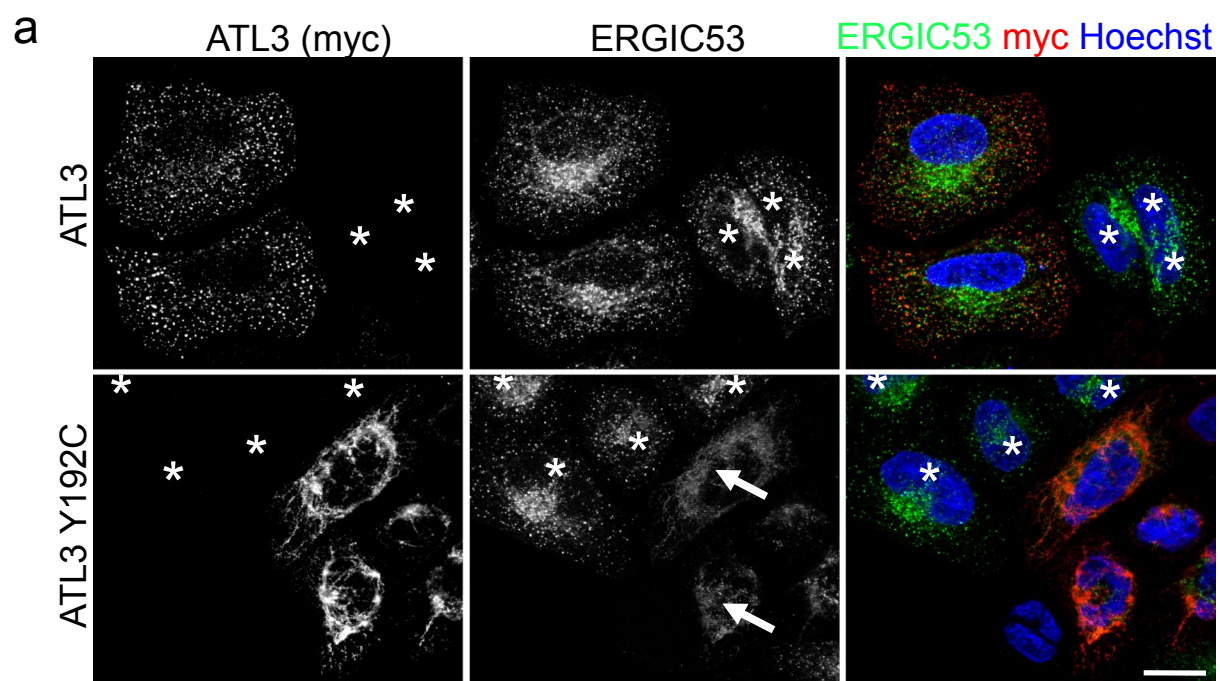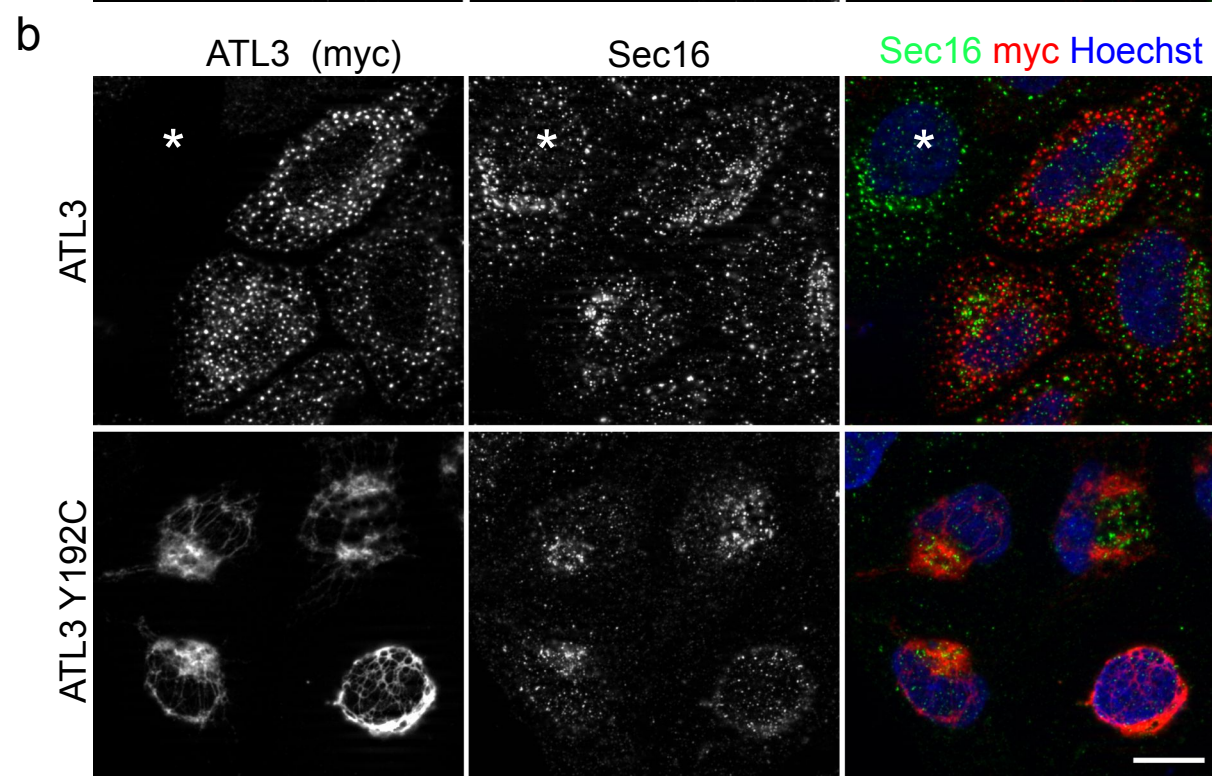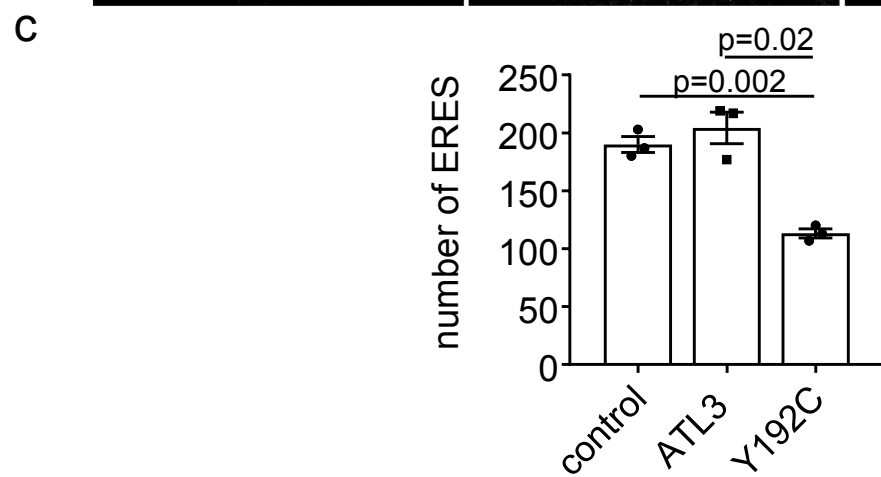

Fig. S3

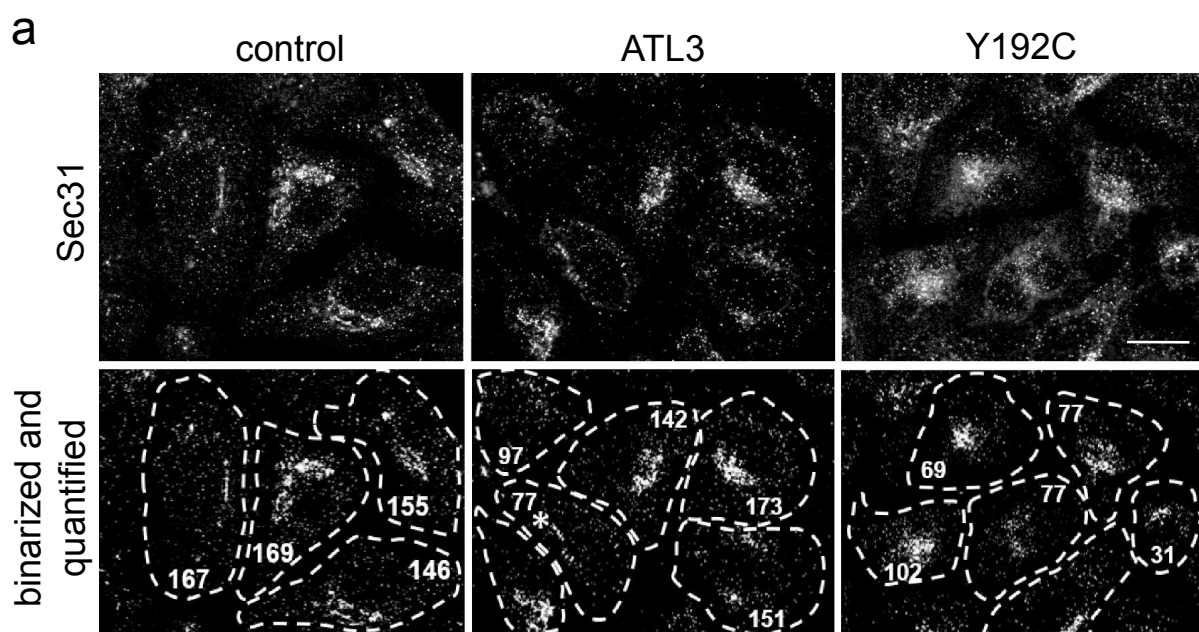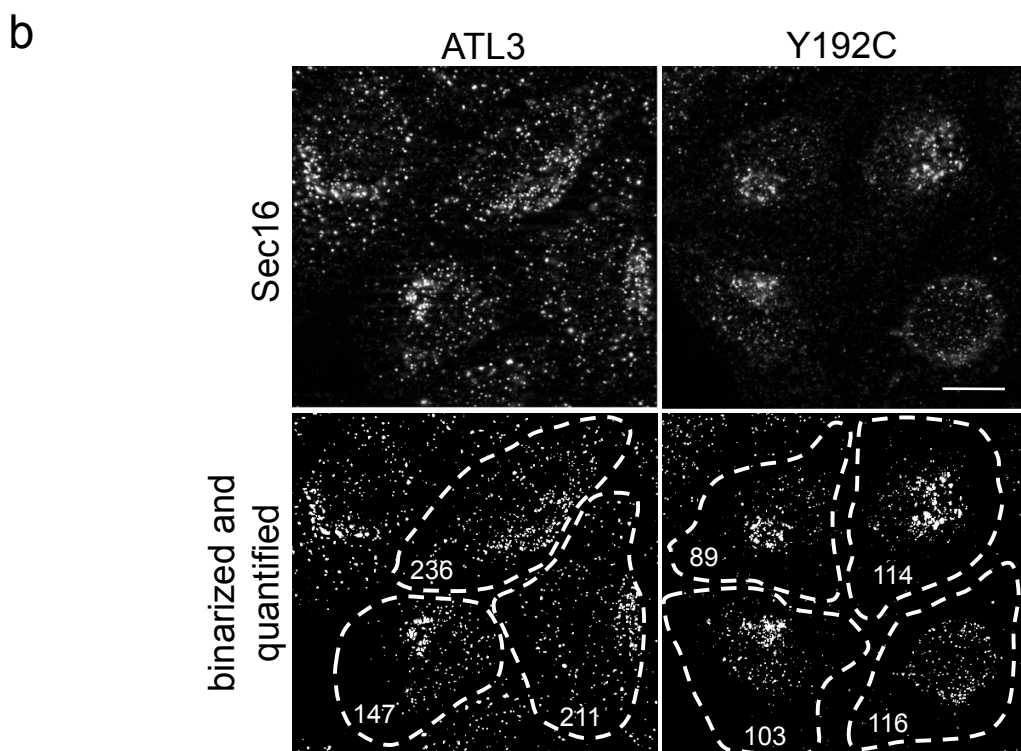

Fig. S4

a

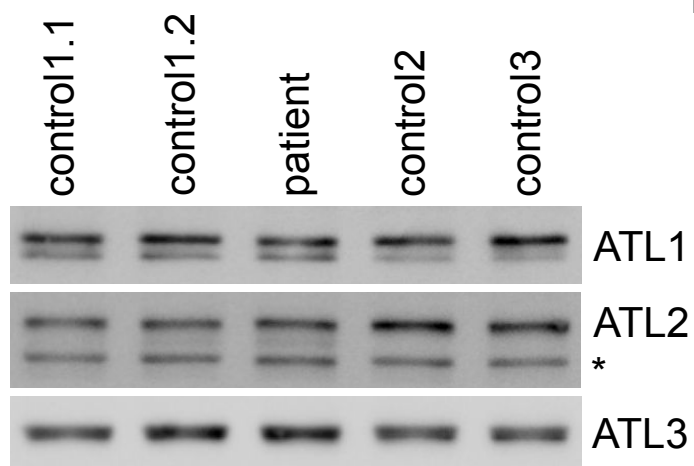

b

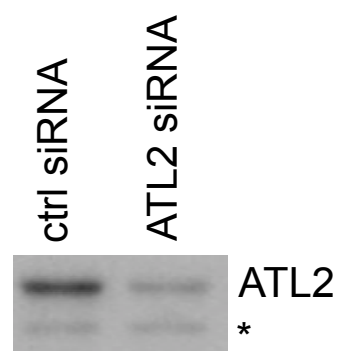

Fig. S5

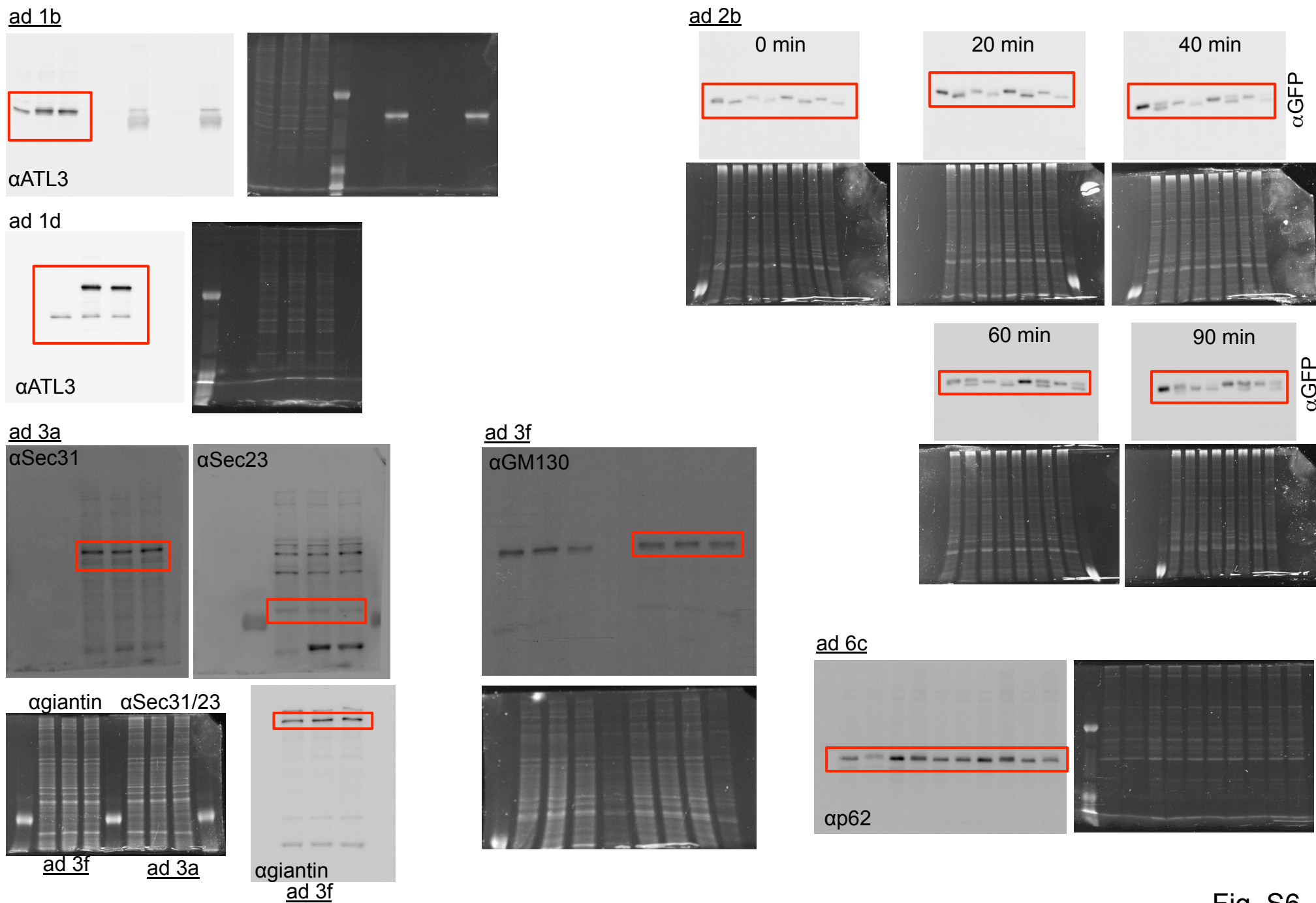

Fig. S6

ad S1b

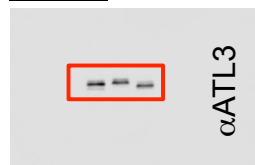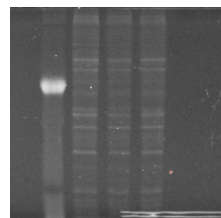

ad S2b

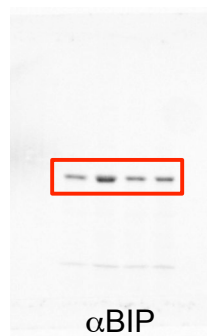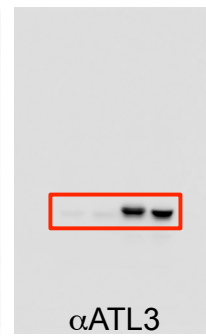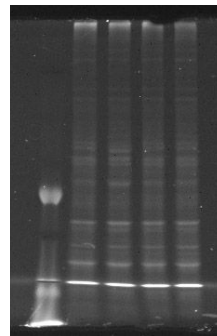

ad S5a

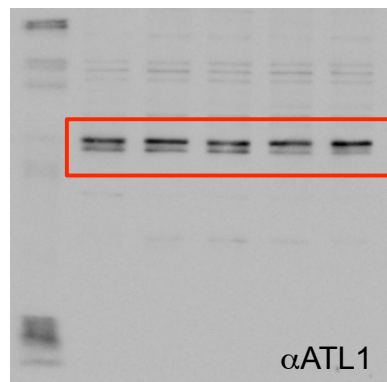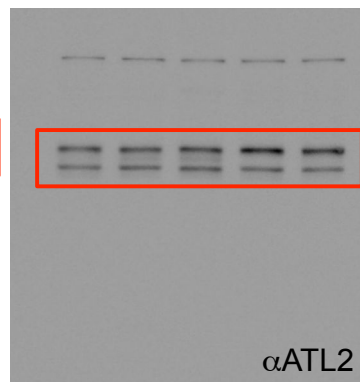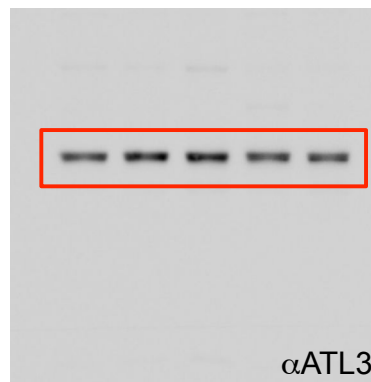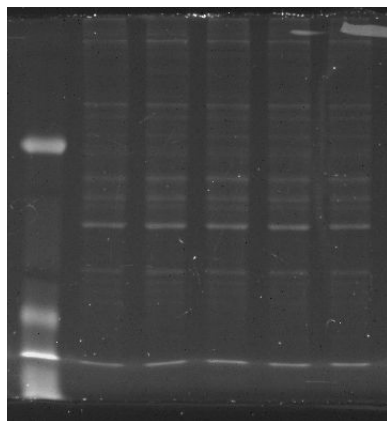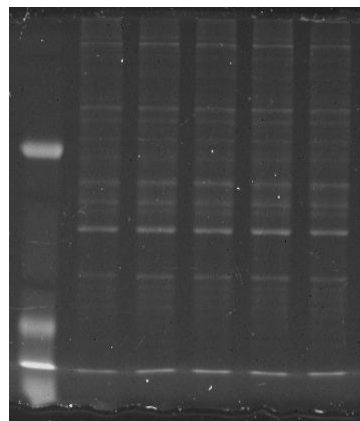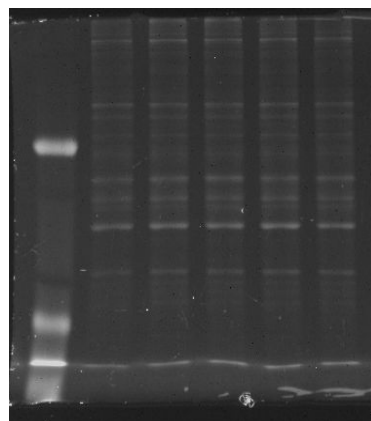

ad S5b

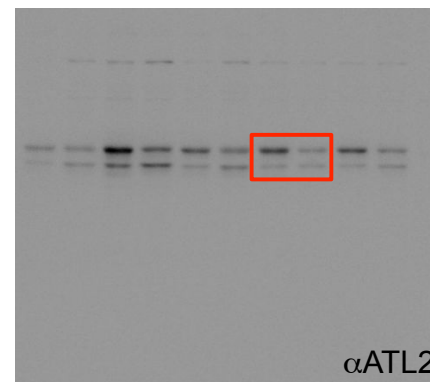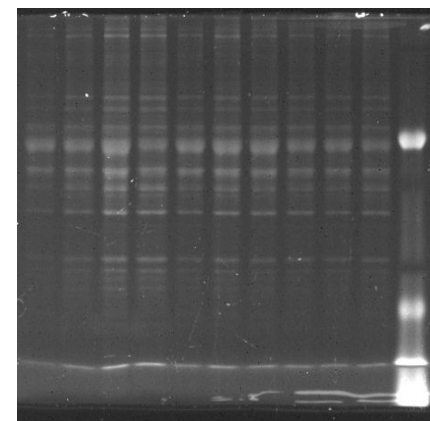

Fig. S7

Supplement: Supplementary file 1 — Supplementary material 1 (PDF 3880 kb) [file 18_2019_3010_MOESM1_ESM.pdf]
