# Supplementary material for: A disease causing ATLASTIN 3 mutation affects multiple endoplasmic reticulum-related pathways
Source: Cell Mol Life Sci. 2019 Jan 21;76(7):1433–45. doi: 10.1007/s00018-019-03010-x (PMC6420906; doi:10.1007/s00018-019-03010-x)
Supplement: Supplementary file 2 — Supplementary material 2 (DOCX 19 kb) [file 18_2019_3010_MOESM2_ESM.docx]

Supplemental experimental information Behrendt et al.

**List of antibodies used**

| **antibody** | **origin,** | **dilution** | **supplier** | **RRID** |
| --- | --- | --- | --- | --- |
| anti-myc | mouse, monoclonal | 1:500 | Santa Cruz (sc-40) | AB_627268 |
| anti-myc | rabbit, polyclonal | 1:500 | Santa Cruz (sc-789) | AB_631274 |
| anti-calnexin | mouse, monoclonal | 1:250 | Chemicon (MAB3126) | AB_2069152 |
| anti-golgin B1 | rabbit, polyclonal | 1:500 (IF)  1:1000 (WB) | Sigma Aldrich (HPA011008) | AB_1079011 |
| anti-sec 31A | mouse, monoclonal | 1:250 (IF)  1:1000 (WB) | Becton Dickinson (612350) | AB_399716 |
| anti-LC3 | rabbit, polyclonal | 1:800 | MBL (PM036) | AB_2274121 |
| anti-lamin A | mouse, monoclonal | 1:500 | Abcam (ab8980) | AB_306909 |
| anti-α-tubulin | mouse, monoclonal | 1:500 | Sigma Aldrich (T6199) | AB_477583 |
| anti-tau | mouse, monoclonal | 1:500 | Chemicon (Mab3420) | AB_94855 |
| anti-sec16 | rabbit, polyclonal | 1:200 | Abcam (ab70722) | AB_1270588 |
| anti-ERGIC53 | mouse, monoclonal | 1:250 | Axxora (ALX-804-602-C100) | AB_2051363 |
| anti-MAP2 | mouse, monoclonal | 1:500 | Sigma-Aldrich (M4403) | AB_477193 |
| anti-Reticulon4 | rabbit, polyclonal | 1:200 | Abcam (ab47085) | AB_881718 |
| anti-ATL3 | rabbit, polyclonal | 1:750 | Abcam (ab104262) | AB_10712136 |
| anti-GM 130 | mouse, monoclonal | 1:250 | BD Bioscience (610822) | AB_398141 |
| anti-GFP | mouse, monoclonal | 1:1000 | Clontech (632381) | AB_2313808 |
| anti-sec31A | mouse, monoclonal | 1:1000 | Becton Dickinson (612350) | AB_399716 |
| anti-sec23 | rabbit, polyclonal | 1:2000 | Bertrand Kleizen, Utrecht University |  |
| anti-ATL2 | rabbit, polyclonal | 1:1000 | Proteintech Europe (16688-1-AP) | AB_1850898 |
| anti-ATL1 | rabbit, polyclonal | 1:1000 | st John's Laboratory (ABIN1876490) |  |
| anti-p62 | mouse, monoclonal | 1:1000 | Abnova ( H00008878-M01) | AB_437085 |
| anti-Bip | goat, polyclonal | 1:1000 | Santa Cruz (sc-1051) | AB_2119994 |
| anti-goat IgG, HRP conjugate | donkey | 1:5000 | Santa Cruz (sc-2020) | AB_631728 |
| anti-rabbit IgG, HRP conjugate | goat | 1:5000 | Promega (W4011) | AB_430833 |
| anti-mouse IgG, HRP conjugate | goat | 1:5000 | Promega (W4021) | AB_430834 |
| Alexa Fluor® 555 anti-mouse | goat | 1:500 | Life Technologies (A-21424) | AB_141780 |
| Alexa Fluor® 555 anti-rabbit | goat | 1:500 | Life Technologies (A-21429) | AB_141761 |
| Alexa Fluor® 488 anti-mouse | goat | 1:500 | Life Technologies (A-11029) | AB_138404 |
| Alexa Fluor® 488 anti-rabbit | goat | 1:500 | Life Technologies (A-11034) | AB_2576217 |
| Alexa Fluor® 647 anti-rabbit | goat | 1:250 | Molecular Probes (A21245) | AB_141775 |

**List of plasmids used**

| **insert** | **vector backbone** | **source/reference** |
| --- | --- | --- |
| pplss-mRFP-KDEL | pEGFP-C1 | kindly provided by Erik Snapp [56] |
| hATL3-myc | pCI | [11] |
| hATL3 Y192C-myc | pCI | [11] |
| VSVG-EYFP | pcDNA3.1 (+) | [31] |
| Sar1b-H79G | pSG5 | kindly provided by Rainer Pepperkok |
| hTERT | pCDH | hTERT from Cagatay Günes cloned into pCDH via EcoRI/BamHI. |
| empty | pcDNA3.1 (+) | Invitrogen (V790-20) |
| GFP-hATL3 | pEGFP-C1 | see materials and methods |
| GFP-hATL3 Y192C | pEGFP-C1 | see materials and methods |
| tomato-KDEL (pplss-tdTomato-KDEL) | pEGFP-C1 | analogous to pplss-mRFP-KDEL, kindly provided by Christina Valkova |
| myc-hATL3 | pcDNA3.1 (+) Hygro | see materials and methods |
| myc-hATL3 Y192C | pcDNA3.1 (+) Hygro | see materials and methods |
| hATL3 | pcDNA3.1 (+) Hygro | see materials and methods |
| hATL3 Y192C | pcDNA3.1 (+) Hygro | see materials and methods |

**List of chemical compounds used**

| **compound** | **supplier** |
| --- | --- |
| Chloroquine | Sigma-Aldrich (C6628) |
| Tunicamycin | Sigma-Aldrich (T7765) |
| Brefeldin A | Sigma-Aldrich (B7651) |
